# Incisional Blepharoplasty with Skin Excision for Upper Eyelid Epiblepharon Correction: Functional and Aesthetic Outcomes in Two Post-Childhood Cases

**DOI:** 10.3390/life16091386

**Published:** 2026-08-22

**Authors:** Shinjiro Kono, Motohiro Kamei

**Affiliations:** Department of Ophthalmology, Aichi Medical University Hospital, Nagakute 480-1103, Japan

**Keywords:** epiblepharon, upper eyelid, excess skin, incisional double-eyelid blepharoplasty, case report

## Abstract

Background: Epiblepharon is characterized by a fold of skin that stretches horizontally across the upper or lower eyelid and is usually associated with eyelash inversion. Upper eyelid epiblepharon is relatively rare and is treated with double-eyelid surgery using a buried suture or an incisional method. To date, no reports have described a detailed treatment course for epiblepharon that simultaneously achieves aesthetic improvement and corrects eyelash inversion. Case presentations: We present the treatment outcomes of two cases of upper eyelid epiblepharon with excess skin in a 16-year-old female adolescent patient and a 35-year-old women, both treated with incisional double-eyelid surgery combined with skin excision. In both patients, eyelash inversion was corrected, double eyelids were formed, and aesthetic improvements were made. Conclusions: By accurately performing appropriate skin excision and suture placement to create double eyelids, a functionally and aesthetically satisfactory surgical outcome can be achieved in patients with upper eyelid epiblepharon.

## 1. Introduction

Epiblepharon is characterized by a fold of skin that stretches horizontally across the upper or lower eyelid and is usually associated with eyelash inversion [1,2,3,4]. It occurs more commonly in the lower eyelid, and documented cases of upper eyelid epiblepharon are relatively rare [4,5,6,7].

Epiblepharon of the upper eyelid is treated with double-eyelid surgery using the buried suture or incisional method [4,5,6,7,8,9,10,11]. Buried suture techniques are commonly used to correct upper eyelid epiblepharon in cases with minimal tissue under the skin of the upper eyelid and in those where eyelash inversion can be easily corrected solely through the formation of a double eyelid crease [4,5,6,7]. If double eyelid disappearance or poor correction of inversion is expected with the buried suture method, incisional double-eyelid surgery is indicated [8,9,10,11]. Furthermore, if the epicanthal folds are prominent, epicanthoplasty is sometimes performed either simultaneously or as an isolated procedure [8,10].

There have been several reports on the use of the buried suture method or epicanthoplasty for epiblepharon of the upper eyelid, with most focusing on surgery performed during childhood [4,5,6,7,8,9,10,11]. However, no report has detailed the course of treatment for upper eyelid epiblepharon with excess skin after childhood to achieve both functional and aesthetic improvements. By creating double eyelids with an appropriate amount of skin excision, unpleasant symptoms caused by the eyelashes are eliminated, visual function is improved, and satisfactory cosmetic results are achieved.

In this report, we present the treatment outcomes of two cases of epiblepharon with excess skin in 16- and 35-year-old women who underwent incisional double-eyelid surgery combined with skin excision. Written informed consent was obtained from the patients for publication of this case report and any accompanying images.

## 2. Detailed Case Description

### 2.1. Case Presentation

#### 2.1.1. Case 1

**Preoperative Assessment**

A 16-year-old female adolescent patient presented with a foreign body sensation in the upper eyelid caused by eyelash inversion. The patient had no previous history of eyelid surgery. She attempted self-correction using eyelash glue; however, she subsequently developed dermatitis, making correction difficult. She sought surgical treatment to achieve a definitive resolution. Visual dysfunction due to thick excess skin was noted, in addition to eyelash inversion of the upper eyelid and corneal epithelial damage, leading to a diagnosis of upper eyelid epiblepharon with excess skin. After gently pinching the excess skin and accurately measuring the margin reflex distance-1, the value was 5.0 mm on both sides, and there was no concomitant ptosis. In this report, “thick excess skin” is defined as redundant anterior upper eyelid tissue—comprising both thick dermis and underlying hypertrophic orbicularis oculi muscle with subcutaneous fat—that physically overhangs the eyelid margin, mechanically prevents the outward rotation of the eyelashes, and requires full-thickness en bloc excision to achieve a stable, functional double-eyelid crease.

**Surgical Planning**

Considering the buried method was expected to result in postoperative under-correction and recurrence, incisional double-eyelid blepharoplasty with excess skin removal was planned and performed at the Aichi Medical University Day Surgery Center for Ophthalmology.

**Surgical Technique**

**Figure 1:**
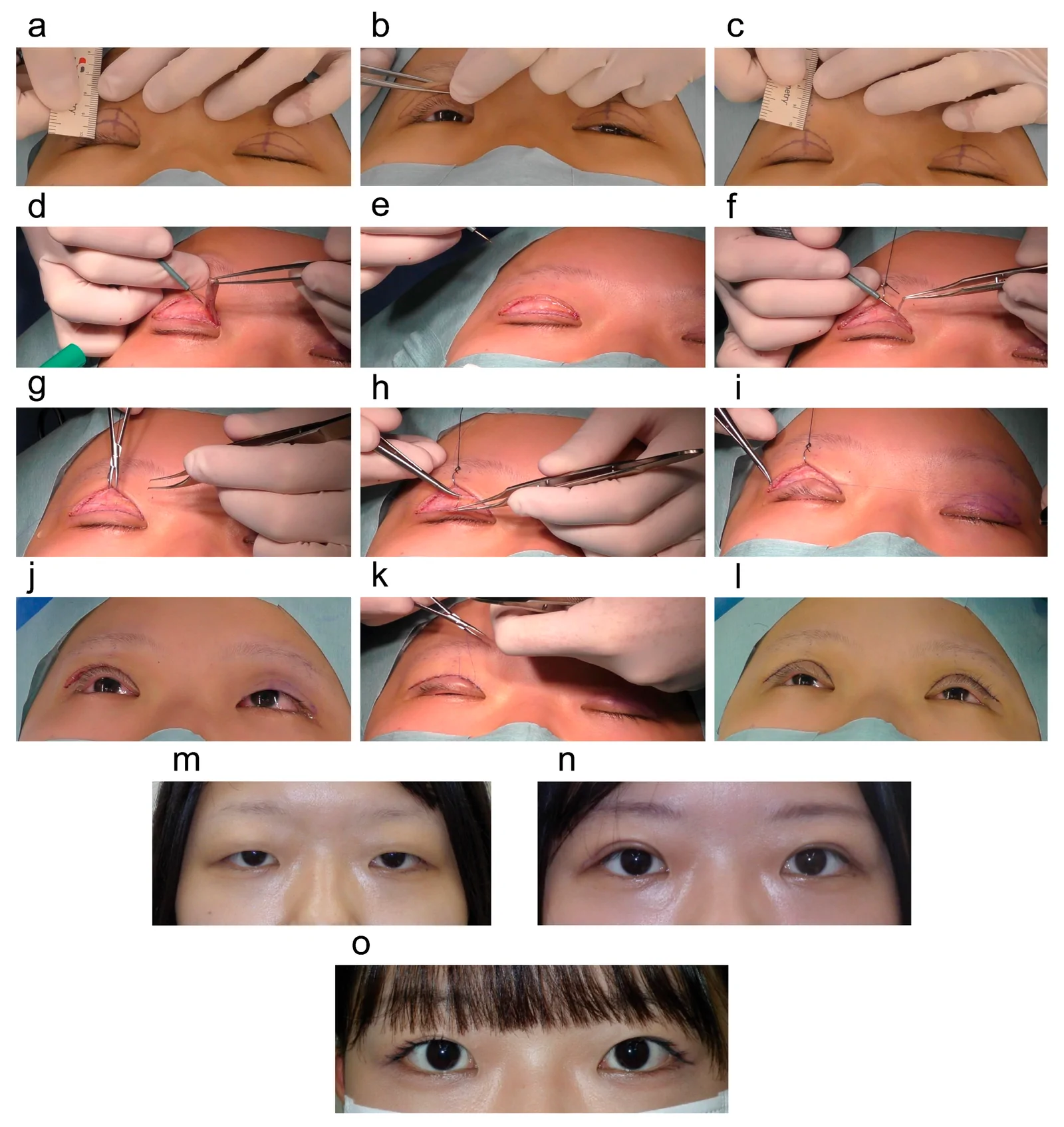
Case 1: Surgical procedure and outcome. (**a**) The incision line was marked to create a double-eyelid crease 7 mm from the eyelid margin. (**b**) The excess skin was measured by pinching it, and the excision area was designed. (**c**) The upper incision line was 14 mm below the eyebrow. (**d**) Using a radiofrequency cutting device, excess skin—including the orbicularis oculi muscle—was excised according to the design. (**e**) Following skin removal, the boundary between the levator aponeurosis and the orbicularis oculi muscle was clear. (**f**) The orbicularis oculi muscle protruding from the eyelid margin was resected. (**g**–**i**) Sutures were passed through the tarsal plate to the subcutaneous tissue of the eyelid margin at six equidistant points. (**j**) The eyelashes rotate outward, and the amount of excised skin was appropriate. (**k**) The skin was closed, and the surgery on the right eyelid was complete. (**l**) The completed surgery. (**m**) Preoperatively, thick excess skin and eyelash inversion were observed. (**n**) One month postoperatively, the excess skin is resolved, a double eyelid is formed, and the eyelashes rotate outward. (**o**) Nine months postoperatively, the swelling has subsided, resulting in a more refined appearance. The double-eyelid width had slightly diminished, although eyelash inversion was absent, and the patient was satisfied.

A parallel incision was created 7 mm from the eyelid margin (Figure 1a). The excess skin was quantified using the pinch technique (Figure 1b). An upper incision line was created approximately 14 mm below the eyebrows (Figure 1c), and a skin excision area was designed. Skin incisions were made following the design, and excess skin—including the orbicularis oculi muscle—was excised (Figure 1d,e).

To avoid interference with the outward rotation of the eyelashes, a portion of the orbicularis oculi muscle at the eyelid margin was excised (Figure 1f). For double-eyelid formation, sutures were passed through the tarsal plate to the subcutaneous tissue of the eyelid margin at six equidistant points (Figure 1g–i). During the eyelid opening, excess skin was prevented from overlapping with the double-eyelid line, and the double eyelid was neatly formed (Figure 1j). The skin was sutured, and the surgery was completed (Figure 1k,l).

**Follow-up and Outcomes**

Postoperatively, the eyelash inversion was corrected, and the swelling gradually subsided. At the 1-month follow-up, the double eyelid appeared more defined, and the patient found it easier to lift her eyelids compared to before the surgery (Figure 1m,n). At 9 months postoperatively, the double-eyelid line had become slightly lower than anticipated; however, the eyelashes did not contact the cornea, and no corneal epithelial damage was observed (Figure 1o). The patient was satisfied with both the aesthetic and functional outcomes. Although further skin excision could have been performed to make the double-eyelid line more distinct for aesthetic purposes, the patient did not request additional surgery because the primary objectives of correcting the eyelash inversion and improving the eyelid heaviness were achieved.

#### 2.1.2. Case 2

**Preoperative Assessment**

A 35-year-old woman with a history of persistent congenital epiblepharon since childhood regularly plucked her upper eyelashes because they curled into her eyes. She was referred to our hospital for surgical treatment and was diagnosed with epiblepharon of the upper eyelid with excess skin. During examination, excess skin laxity with lateral hooding and corneal epithelial damage due to eyelash inversion were observed. After gently pinching the excess skin and accurately measuring the margin reflex distance-1, the value was 5.0 mm on both sides, and there was no concomitant ptosis. She had no previous history of eyelid surgery.

**Surgical Technique**

**Figure 2:**
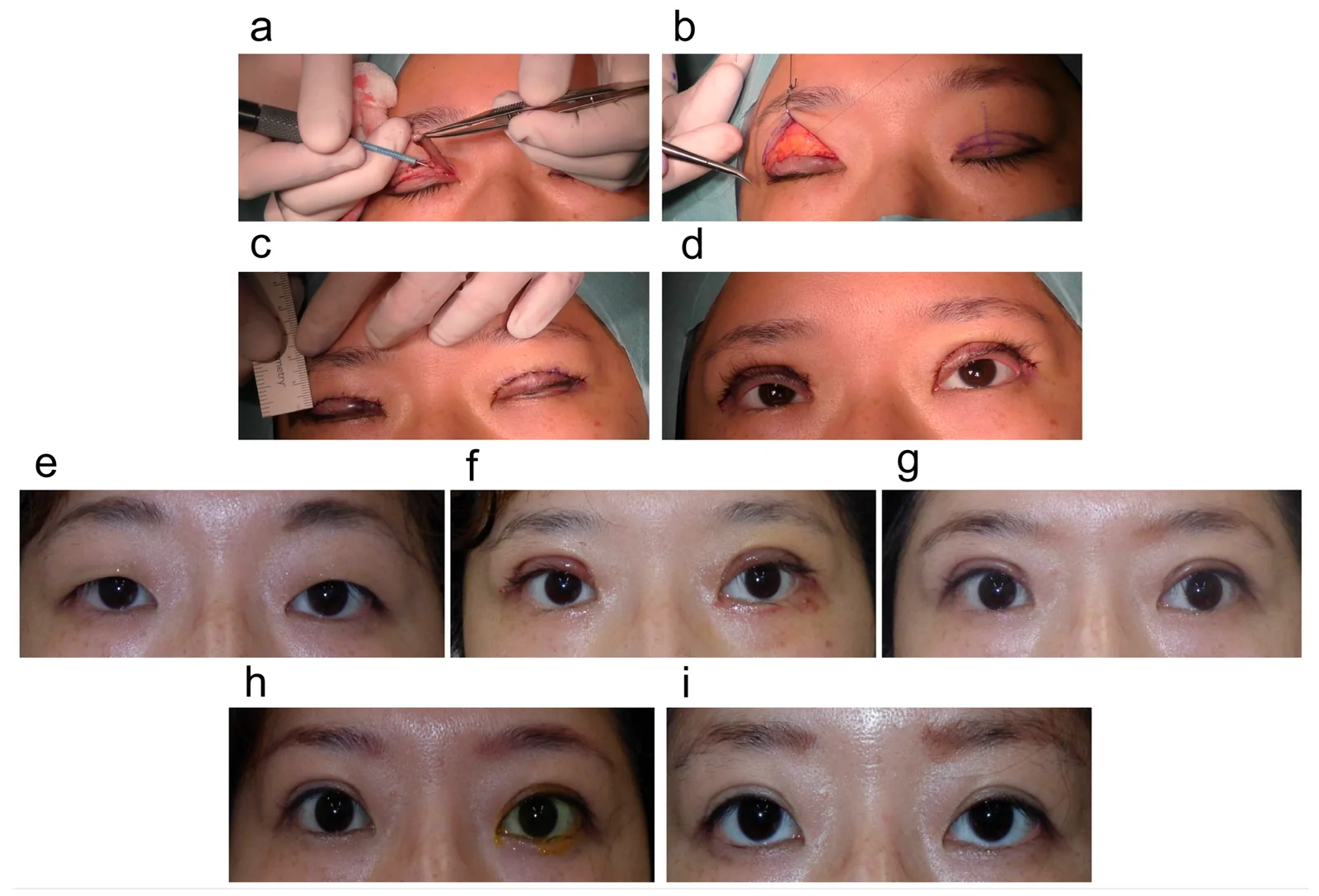
Case 2: Surgical procedure and outcome. (**a**) As in Case 1, after making an incision along the design, the excess skin—including the orbicularis oculi muscle—was excised. (**b**) The thread was passed from the tarsal plate to the eyelid margin to create a double eyelid. (**c**) Double-eyelid surgery was performed on both sides, and the incisions were closed. The distance between each eyebrow and the double-eyelid crease was measured to confirm the absence of asymmetry. (**d**) Immediately postoperatively, the eyelashes rotated outward, and there was no asymmetry in the double eyelids. (**e**) Preoperatively, excess skin and eyelash inversion are present. (**f**) One week postoperatively, some swelling is present; however, the double eyelid had formed, and eyelashes rotate outward. (**g**) One month postoperatively, the double-eyelid width became appropriate, and the eyelashes rotate outward. (**h**) Six months postoperatively. (**i**) At 12 months postoperatively, the double-eyelid width is unchanged, and visual function is maintained.

The surgery was completed using the same procedure as in Case 1. After removing the excess skin, including the orbicularis oculi muscle, six double-eyelid sutures were placed, and a double-eyelid crease line was designed 7 mm from the eyelid margin (Figure 2a,b). Considering the excess lateral skin was excised more widely compared to the medial skin, the distance between the eyebrows and the double eyelid after excess skin excision was 15 mm over the medial canthus and 14 mm over the lateral canthus (Figure 2c,d).

**Follow-up and Outcomes**

**Figure 3:**
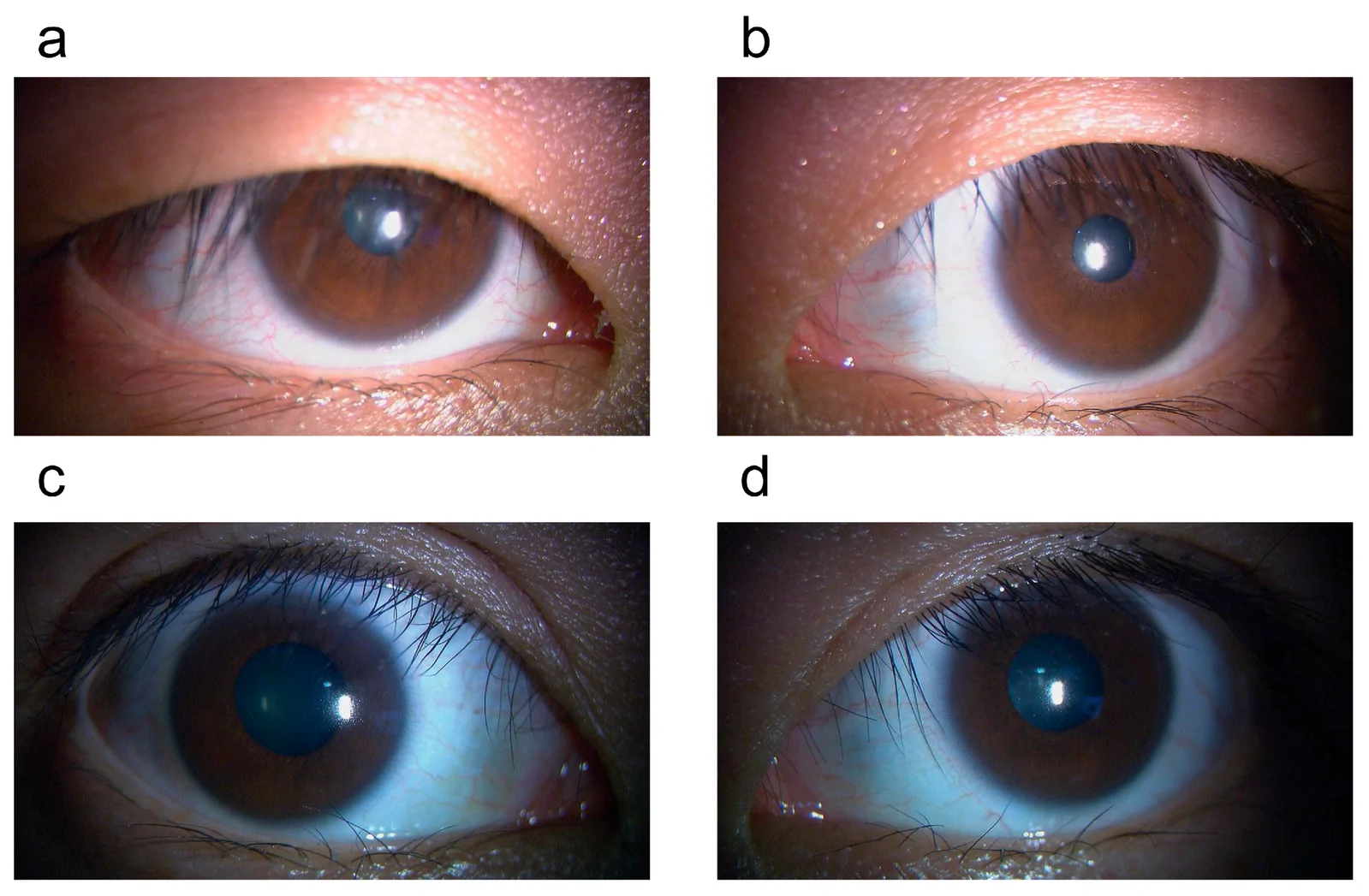
Case 2: Slit-lamp microscopy image. (**a**,**b**) Preoperatively, the eyelashes are drooping and touching the cornea. (**c**,**d**) Twelve months postoperatively, double eyelids have formed, and the eyelashes are not touching the cornea.

Immediately postoperatively, the eyelash inversion was corrected, and no corneal epithelial damage was observed (Figure 2e–i and Figure 3a–d). One year postoperatively, the double eyelid was formed as anticipated and maintained (Figure 2i). The visual function and appearance were improved, and the patient was satisfied with the surgical outcome.

## 3. Discussion

We treated two patients with upper eyelid epiblepharon with excess skin and eyelash inversion. After surgery, the eyelash inversion was corrected, and the eyelids were lifted sufficiently, providing good vision. In cases of upper eyelid epiblepharon where excess skin is minimal and the upper eyelid is thin during early childhood, the buried suture method is effective. In recent years, technical refinements aimed at maintaining double eyelids have been reported [4,7]. However, to the best of our knowledge, no study has focused on the treatment outcomes of epiblepharon in patients with thick excess skin after childhood. Although incisional double-eyelid surgery carries the risk of postoperative eyelid margin scarring, it enables the reliable placement of multiple sutures to form a double fold, making it effective for maintaining the double crease and correcting eyelash inversion [4,8,11].

In the two cases presented here, the double-eyelid line was designed to be slightly high. Sutures were accurately passed through the tarsal plate and subcutaneous tissue to create the double-eyelid line, and excess skin quantified by the pinch technique was excised. The eyelash inversion correction resulted in satisfactory postoperative outcomes, both aesthetically and functionally. The epicanthal fold was inconspicuous, and epicanthoplasty was unnecessary. For those born with single eyelids, considering the tendency for the double-eyelid width to revert after surgery due to the original eyelid weight and thickness, creating a double-eyelid width of ≥7 mm is desirable.

Few studies have examined practical widths regarding the distance between the eyebrow and the eyelid margin, or the double eyelid width, in blepharoplasty. One recent report recommends a ratio of approximately 1:2 for the distance between the double-eyelid crease and the eyelid margin and that between the eyebrow and the double-eyelid crease to achieve aesthetic outcomes; this ratio was also appropriate in our cases [12]. Further skin excision carries the risk of creating an unnatural appearance due to an excessively wide double-eyelid crease. It is preferable to limit excision such that only a slight amount of excess skin overlaps with the double-eyelid line.

To achieve high patient satisfaction not only for those seeking double-eyelid surgery for purely cosmetic reasons but also for patients requiring correction of upper eyelid epiblepharon, it is recommended to perform the procedure while considering the distance ratios between the brow, the double-eyelid crease, and the eyelid margin. Further accumulation of clinical cases is necessary to determine the precise proportional relationships tailored to individual patients.

In the present cases, the incision scars were hidden by the double eyelids. However, the scar area—where the lateral skin is widely removed—can sometimes be visible, which may be an aesthetic concern. Nonetheless, when sutures are accurately placed, scarring becomes less noticeable over time in most cases. In the two cases in the current report, the incision scars did not pose a problem.

This study had some limitations warranting acknowledgment. First, the sample size is small (*n* = 2), which restricts the immediate generalizability of our findings to all epiblepharon cases. However, while buried suture methods carry a high risk of recurrence in post-childhood patients with thick redundant skin, the complete resolution of corneal erosion, freedom from eyelash adhesives, and stable fold formation achieved in these cases demonstrate the practical utility of this tailored incisional approach as a valuable surgical option for carefully selected patients. Second, the follow-up period was 9–12 months; therefore, longer-term follow-up is desirable to confirm the degree of eyelash correction and the maintenance of double-eyelid formation. Third, the individuality of skin thickness and texture may influence the degree of postoperative double-eyelid formation, necessitating further investigation.

## 4. Conclusions

We report two cases of upper eyelid epiblepharon in patients with thick excess skin. By accurately performing appropriate skin excision and suture placement to create double eyelids, satisfactory functional and aesthetic surgical outcomes can be achieved. Further studies with larger patient populations are needed to confirm the long-term stability and broader applicability of this technique.

## Data Availability

The data that support the findings of this study are not publicly available due to privacy reasons but are available from the corresponding author, S.K., on reasonable request.
